# First Evidence of the Expression and Localization of Prothymosin α in Human Testis and Its Involvement in Testicular Cancers

**DOI:** 10.3390/biom12091210

**Published:** 2022-08-31

**Authors:** Massimo Venditti, Davide Arcaniolo, Marco De Sio, Sergio Minucci

**Affiliations:** 1 Dipartimento di Medicina Sperimentale, Sez. Fisiologia Umana e Funzioni Biologiche Integrate “F. Bottazzi”, Università degli Studi della Campania “Luigi Vanvitelli”, Via Costantinopoli, 16-80138 Napoli, Italy; 2 Dipartimento della Donna, del Bambino e di Chirurgia Generale e Specialistica, Università degli Studi della Campania “Luigi Vanvitelli”, Via Luigi De Crecchio, 02-80138 Napoli, Italy

**Keywords:** prothymosin α, testis cancer, classic seminoma, Leydig cell tumor, apoptosis, autophagy

## Abstract

Prothymosin α (PTMA) is a phylogenetically conserved polypeptide in male gonads of Vertebrates. In Mammals, it is a ubiquitous protein, and, possessing a random-coil structure, it interacts with many other partners, in both cytoplasmic and nuclear compartments. PTMA has been widely studied during cell progression in different types of cancer because of its anti-apoptotic and proliferative properties. Here, we provided the first evidence of PTMA expression and localization in human testis and in two testicular cancers (TC): classic seminoma (CS) and Leydig cell tumor (LCT). Data showed that its protein level, together with that of proliferating cell nuclear antigen (PCNA), a cell cycle progression marker, increased in both CS and LCT samples, as compared to non-pathological (NP) tissue. Moreover, in the two-cancer tissue, a decreased apoptotic rate and an increased autophagic flux was also evidenced. Results confirmed the anti-apoptotic action of PTMA, also suggesting that it can act as a switcher from apoptosis to autophagy, to favor the survival of testicular cancer cells when they develop in adverse environments. Finally, the combined data, even if they need to be further validated, add new insight into the role of PTMA in human normal and pathological testicular tissue.

## 1. Introduction

Prothymosin α (PTMA) is a small polypeptide with extremely peculiar features. Indeed, the lack of cysteines, methionine, and aromatic amino acids, as well as the presence, in its primary structure, of almost 50% of aspartic and glutamic residues [1], makes PTMA one of the most acidic proteins present in nature (isoelectric point 3.5). Moreover, the absence of secondary or tertiary structures [2] not only allows PTMA to interact with many cationic molecules but also to acquire transitional, biologically active conformations, depending on the molecule with which it interacts.

This defines the functional promiscuity of PTMA, also reflected by its ubiquitous localization in the nucleus (because of the presence of a bipartite nuclear localization signal at the C-terminal), in the cytoplasm, as well as in the extracellular compartment [3,4].

If, on one hand, the cytoplasmic role of PTMA is far from being completely understood, many reports demonstrated its crucial role for survival and proliferation of normal and cancer cells [5,6], such as squamous cell carcinoma of the breast [7], head and neck [8], hepatocellular carcinoma [9,10], lung cancer [10,11], gallbladder cancer [12], colorectal cancer [13], etc.

PTMA prevents apoptosis from inhibiting apoptosome formation [14], controls gene expression and chromatin remodeling through histone H1 binding [15,16,17,18] and, via its association with the oncogene *c-myc*, it favors cell cycle progression [19]. Moreover, in diverse human tissues, and besides the regulative role in cellular processes such as cell proliferation, chromatin remodeling, and transcription, PTMA also seems to be involved in the expression of oxidative stress response genes, and, consequently, also in the autophagic pathway, potentiating repair mechanisms to allow the degradation of damaged organelles/molecules [20,21].

PTMA was usually supposed to be a protein exclusive to mammals [22], but, in 2002, our group cloned a cDNA coding for PTMA for the first time in the testis of a non-mammalian vertebrate, the frog *Pelophylax esculentus*, showing changes during the annual spermatogenic cycle in its expression and localization, suggesting for a specific role in the frog testicular activity [23,24]. Later, its association with the differentiative phase of spermatogenesis has been also proved in other species [25], such as *Torpedo marmorata* [26], *Danio rerio* [27], *Rattus norvegicus* [28], as well as in rat and human sperm [29,30]. Nevertheless, the involvement of PTMA in testicular cancers (TC) has not been determined yet.

TC, although extremely rare in the general men population, represents one of the most common solid tumors in adolescents and young adult males (between 20 and 40 years) [31,32,33,34]. The etiology of these cancers remains largely unclear, though epidemiological and clinical studies demonstrated the relationship between TC insurgence and many risk factors, such as age, body size, other pathologies (cryptorchidism, genetic abnormalities, sub-infertility), family, and personal history of TC [35]. Moreover, the increased incidence of TC in the most industrialized countries raised the hypothesis of correlation with environmental pollutants exposure, including endocrine-disrupting chemicals [36,37], whose ability to disturb the physiological hypothalamus/hypophysis/testis axis is broadly recognized, with deleterious effects on spermatogenesis [38].

TC is a general definition of an extremely heterogeneous pathology, including several types of cancer, such as germ cell tumors (GCT), sex cord–gonadal stromal tumors (SCGST), and secondary testicular tumors that are, in turn, classified and divided into different subgroups by the International Agency for Research in Cancer of the World Health Organization (WHO) [39].

Thus, the purpose of this study was to evaluate, for the first time, PTMA expression and localization in human testis, not only in normal (non-pathological (NP) tissue, but also in two of the most common solid tumors in the adolescent and young adult male population (between 20 and 40 years old), namely the classic seminoma (CS) and the Leydig cell tumor (LCT) [31,32,33,34].

## 2. Materials and Methods

### 2.1. Tissue Samples

Testicular tissues biopsies were performed during inguinal exploration in patients with a suspect testis tumor based on clinical examination and ultrasonography. Biopsy was carried out from the suspect testicular lump, and the presence of cancer was confirmed with extemporaneous evaluation by an expert pathologist [40]. The number of collected samples was 5 NP, 10 CS, and 4 LCT. A written informed consent was obtained from all subjects involved in the study. Each tissue sample was then cut into two halves: one half was quickly immersed in liquid nitrogen and stored at −80 °C for Western blot (WB) analysis, and another one was fixed in 10% formalin for histochemical investigations. The study was conducted according to the guidelines of the Declaration of Helsinki and approved by the Ethics Committee of “Università degli Studi della Campania Luigi Vanvitelli” (protocol code 206 approved on 15 April 2019).

### 2.2. Total Protein Extraction and WB Analysis

The testicular tissue samples were lysed in radioimmunoprecipitation assay (RIPA) lysis buffer (#TCL131; Hi Media Laboratories GmbH; Einhausen, Germany) supplemented with 10 µL/mL of protease inhibitors mix (#39102; SERVA Electrophoresis GmbH; Heidelberg, Germany). The homogenates were sonicated by three strokes (20 Hz for 20 s each); after centrifugation for 30 min at 10.000 g, the supernatants were stored at −80 °C [41]. Lowry assay was used to measure protein concentration [42]. A total of 40 µg of proteins was separated by 15% SDS-polyacrylamide gel electrophoresis and transferred to Hybond-P polyvinylidene fluoride membranes (Amersham Pharmacia Biotech, Buckinghamshire, UK) at 280 mA for 2.5 h at 4 °C. The filters were treated for 2 h with blocking solution (5% skim milk in Tris-buffered saline (TBS; 10 mM Tris–HCl pH 7.6, 150 mM NaCl)) containing 0.25% Tween-20 (Sigma–Aldrich Corp., Milan, Italy) before the addition of anti-PTMA (#ab247074; Abcam, Cambridge, UK), anti-proliferating cell nuclear antigen (PCNA; #MA5-11358; Thermo Fisher Scientific, Waltham, MA, USA), anti-B-cell lymphoma 2 (Bcl-2; #E-AB-60012; Elabscience Biotechnology, Wuhan, China), anti-Bcl-2 Associated X (Bax; #E-AB-13814; Elabscience Biotechnology, Wuhan, China), anti-Caspase-3 (#E-AB-22115; Elabscience Biotechnology, Wuhan, China), anti-microtubule-associated proteins 1A/1B light chain 3B (LC3B; #L7543; Sigma–Aldrich, Milan, Italy), anti-sequestosome 1 (p62; #E-AB-63539; Elabscience Biotechnology, Wuhan, China), or anti-β-Actin (#E-AB-20031; Elabscience Biotechnology,Wuhan, China) antibodies diluted 1:5000 (for β-Actin) and 1:1000 (for all the others) in the blocking solution and incubated overnight at 4 °C. After three washes in TBST (TBS including 0.25% Tween20), the filters were incubated with horseradish peroxidase-conjugated anti-rabbit immunoglobulin G (IgG; #AP307P; Sigma–Aldrich Corp., Milan, Italy), or anti-mouse IgG (#AP130P; Sigma–Aldrich Corp., Milan, Italy), all diluted 1:10,000 in the same blocking solution. Then, the filters were washed three times again in TBST, and the immunocomplexes were revealed using the enhanced chemiluminescence-WB analysis detection system (Amersham Pharmacia Biotech, Buckinghamshire, UK). All the signals, including those used to obtain the Bax/Bcl-2 and LC3B-II/LC3B-I ratios, were quantified by densitometry analysis using the software ImageJ (version 1.53 g) and adjusted relative to β-Actin levels. All the experiments were performed in triplicate.

### 2.3. Immunofluorescence (IF) Analysis and TUNEL Assay

The fixed tissues were dehydrated in increasing alcohol concentrations before paraffin embedding. The 5 µm thick serial sections were stained with hematoxylin/eosin. Slides were viewed under an optical microscope (Leica DM 2500, Leica Microsystems, Wetzlar, Germany), and photographs were taken using the Leica DFC320 R2 digital Camera.

For IF staining, 5 µm thick testicular tissue sections were deparaffinized and rehydrated, followed by antigen retrieval performed by pressure immersing the slides in 0.01 M citrate buffer (pH 6.0) for 3 min [43,44]. To diminish autofluorescence background, tissues were quenched with 0.3 M glycine in phosphate-buffered saline (PBS) for 30 min; then, permeabilization was obtained by incubating the slides with 0.1% (*v*/*v*) Triton X-100 in PBS for 30 min. Later, non-specific binding sites were blocked with PBS containing 5% bovine serum albumin (BSA) and normal goat serum diluted 1:5, before the incubation with primary antibodies (all diluted 1:100 in the blocking solution) overnight at 4 °C. After two washes in TPBS (PBS containing 0.25% Tween20) and two washes in PBS, the secondary antibodies (anti-rabbit Alexa Fluor 488 (#A32731; Thermo Fisher Scientific, Waltham, MA, USA) and anti-mouse Alexa Fluor 647 (#A21236; Thermo Fisher Scientific, Waltham, MA, USA)) diluted 1:500, and peanut agglutinin (PNA) lectin (1:50) to stain the acrosome (#L32458; Alexa Fluor 568; Thermo Fisher Scientific, Waltham, MA, USA) in the blocking mixture were added for 1 h at RT. Finally, slides were washed again, and the cells’ nuclei were marked with Vectashield+ 4′,6-diamidino-2-phenylindole (DAPI; H-1200-10; Vector Laboratories, Peterborough, UK). The sections were observed and captured with the optical microscope (LeicaDM5000 B + CTR 5000) with a UV lamp and saved with IM 1000 software (version 4.7.0).

Apoptosis was examined by the Terminal deoxynucleotidyl transferase dUTP nick end labeling (TUNEL)-assay using the DeadEnd™ Fluorometric TUNEL System (#G3250; Promega Corp., Madison, WI, USA) following the manufacturer’s protocol. The cell nuclei were marked with DAPI (#MBD0015; Sigma–Aldrich, Milan, Italy). IF and TUNEL-assay experiments have been performed in triplicate (the used images are just representative), and for the fluorescent signal analysis and the count of the percentage of TUNEL positive cells, 20 fields/samples, for a total or 300 NP, 600 CS and 240 LCT have been considered and analyzed.

### 2.4. Statistical Analysis

Data were reported as mean ± standard error (SEM). Differences between the groups were considered statistically significant at *p* < 0.05. Analyses were performed using one-way ANOVA; Tukey’s post hoc *t*-test was applied when appropriate with Prism 5.0, GraphPad Software (version Prism 8.4.2; San Diego, CA, USA).

## 3. Results

### 3.1. PTMA and PCNA Expression and Localization in NP, CS and LCT Testicular Tissue

**Figure 1:**
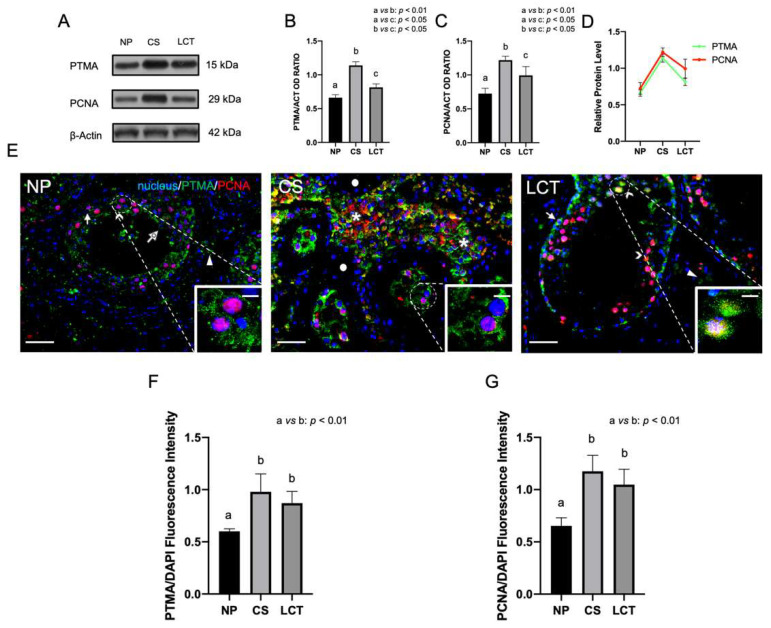
Western blot (WB) and immunofluorescence (IF) analysis of prothymosin α (PTMA) and proliferating cell nuclear antigen PCNA in NP, CS and LCT testicular tissue. (**A**) WB analysis showing the expression of PTMA (15 kDa); PCNA (29 kDa) and β-Actin (42 kDa) in testicular tissue of NP, CS and LCT. (**B**,**C**) histograms showing the relative protein levels of PTMA and PCNA, respectively. Data were normalized with β-Actin and reported as OD ratio. Values are expressed as means ± SEM; (**D**) merged PTMA and PCNA graph in NP, CS and LCT; (**E**) IF analysis of PTMA (green) and PCNA (red) in testicular tissues of NP, CS and LCT. Slides were counterstained with DAPI (blue) which mark the nucleus. Scale bars represent 20 µm and 10 µm in the insets. Arrow: spermatogonia (SPG); arrowheads: spermatocytes (SPC); dotted arrow: spermatids (SPT); triangle: Leydig cells (LC); *: seminoma cells; dot: negative seminoma cells; (**F**,**G**) histograms showing the fluorescence signal intensity of PTMA and PCNA, respectively. Data were normalized with DAPI signal. a vs. b: *p* < 0.01; a vs. c: *p* < 0.05; b vs. c: *p* < 0.05.

WB analyses showed that PTMA was expressed in human NP testicular tissue (Figure 1A,B). Interestingly, its protein level increased in the cancer condition of 72% in CS (*p* < 0.01; Figure 1 A,B) and of 23% in LCT (*p* < 0.05; Figure 1A,B) as compared to NP.

**Figure 2:**
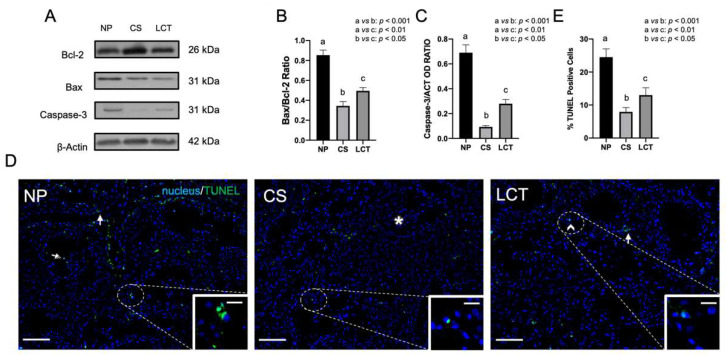
WB analysis of Bcl-2, Bax and Caspase-3 and Terminal deoxynucleotidyl transferase dUTP nick end labeling (TUNEL) assay in NP, CS and LCT testicular tissue. (**A**) WB analysis showing the expression of Bcl-2 (26 kDa); Bax (23 kDa) and Caspase-3 (31 kDa) in testicular tissue of NP, CS and LCT; (**B**,**C**) histograms showing the Bax/Bcl-2 ratio and the relative protein levels of Caspase-3, respectively. Data were normalized with β-Actin and reported as OD ratio. Values are expressed as means ± SEM; (**D**) TUNEL assay in testicular tissues of NP, CS and LCT. Slides were counterstained with DAPI (blue) which mark the nucleus. Scale bars represent 20 µm and 10 µm in the insets. Arrow: SPG; arrowheads: SPC; dotted arrow: SPT; *: seminoma cells. (**E**): a histogram showing the percentage of TUNEL positive cells in NP, CS and LCT samples. a vs. b: *p* < 0.001; a vs. c: *p* < 0.01; b vs. c: *p* < 0.05.

Similarly, and not surprisingly, the protein level of PCNA, a common marker of DNA synthesis and, thus, of cell proliferation, increased 68% in CS (*p* < 0.01; Figure 1A,C) and 37% in LCT (*p* < 0.05; Figure 1A,C) as compared to NP. In fact, in Figure 2D, a similar tendency between PTMA levels and those of PCNA was evidenced, emphasizing that both are likely involved in the enhancement of cancer cell proliferation.

This trend was confirmed by IF analysis. First, a haematoxylin-eosin staining on sections of NP, CS and LCT testicular tissue samples was performed (Figure S1).

In NP, PTMA localized in the cytoplasm of mitotic spermatogonia (SPG; arrow; Figure 1E and inset) and in meiotic spermatocytes (SPC; arrowhead; Figure 1E) cells, with a more intense signal in the latter, as well as in differentiating spermatids (SPT; dotted arrow; Figure 1E). Moreover, a positive PTMA signal was detected also in the interstitial LC (triangle, Figure 1E). Finally, an evident PCNA staining in SPG and SPC nuclei was also observed

Interestingly, in CS, a peculiar PTMA and PCNA signals was seen: indeed, in the cytoplasm and the nucleus of some seminoma cells clusters, their intensity was evident (asterisks; Figure 1E and inset), while in others they were completely absent (dots; Figure 1E). Moreover, the intermediate yellow-orange tint reflected the co-localization of the two proteins in many nuclei.

PTMA localization in the seminiferous epithelium of LCT did not differ from that observed in NP; however, it is worth noting its presence, in co-localization with PCNA; in addition, in the nucleus of the scattered SPC (arrowhead; Figure 1E and inset), it was still present among the abundant tumoral Leydig cells.

Finally, both PTMA and PCNA immunofluorescent signals showed a significant increase in CS and LCT as compared to those of NP (*p* < 0.01; Figure 1F,G).

### 3.2. Apoptotic Rate in NP, CS and LCT Testicular Tissue

To confirm the enhanced proliferation rate in CS and LCT, the apoptotic pathway was also investigated. WB analysis revealed a decreased Bax/Bcl-2 ratio in CS (*p* < 0.01; Figure 2A,B) and LCT (*p* < 0.05; Figure 2A,B) as compared to NP. An opposite trend concerning Caspase-3 protein level was observed, as it decreased in CS (*p* < 0.001; Figure 2A,C) and in LCT (*p* < 0.01; Figure 2A,C) as compared to NP.

To support these data, a TUNEL assay on all the tissues was performed (Figure 2D). Data showed the presence of several apoptotic cells in NP, especially SPG and SPC (Figure 2D and insets). As expected, in both CS (*p* < 0.001; Figure 2D and inset, Figure 2E) and LCT (*p* < 0.01; Figure 2D and inset, Figure 2E) samples, as compared to NP, apoptotic cells were drastically reduced, as just a few dispersed TUNEL-positive cells were still visible.

### 3.3. Autophagy in NP, CS and LCT Testicular Tissue

**Figure 3:**
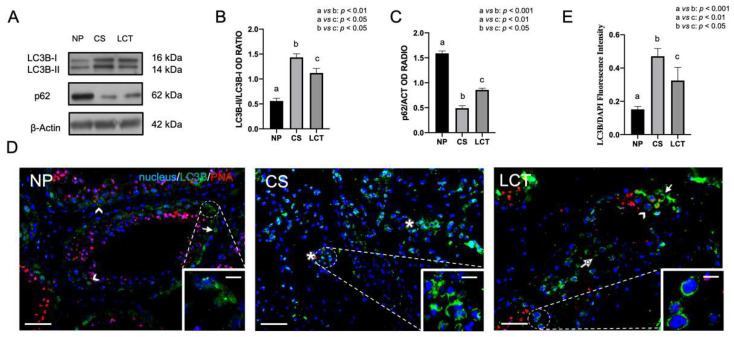
WB and IF analysis of LC3B and p62 in NP, CS and LCT testicular tissue. (**A**) WB analysis showing the expression of LC3B-I (16 kDa); LC3B-II (14 kDa) and p62 (62 kDa) in testicular tissue of NP, CS and LCT; (**B**,**C**) histograms showing the relative protein levels of LC3B-II and p62, normalized, respectively, with LC3B-I and β-Actin and reported as OD ratio. Values are expressed as means ± SEM; (**D**) IF analysis of LC3B (green) in testicular tissues of NP, CS and LCT. Slides were counterstained with PNA (red) which marks the acrosome, and with DAPI (blue), which mark the nucleus. Scale bars represent 20 µm and 10 µm in the insets. Arrow: SPG; arrowheads: SPC; dotted arrow: SPT; *: seminoma cells; (**E**) histogram showing the quantification of LC3B fluorescence signal intensity with respect to DAPI signal. a vs. b: *p* < 0.01 (in B) and *p* < 0.001 (in C,D); a vs. c: *p* < 0.05 (in B) and *p* < 0.01 (in C,D); b vs. c: *p* < 0.05.

A crosstalk between apoptosis and autophagy in normal, cancer, and stressed cells/tissue has been demonstrated [45,46,47,48,49]. Verification of whether an autophagic pathway is activated in TC, LC3B and p62, autophagy markers was also analyzed (Figure 3).

WB analysis showed a significant increase of LC3B-II protein level in both CS (*p* < 0.01; Figure 3A,B) and LCT (*p* < 0.05; Figure 3A,B) as compared to NP.

On the contrary, the p62 protein level showed an opposite behavior, since either in CS (*p* < 0.001; Figure 3A,C) and in LCT (*p* < 0.01; Figure 3A,C) its level decreased as compared to NP, confirming the increased rate of autophagy.

The activation of autophagy was further confirmed by LC3B IF staining, shown in Figure 3D. In NP, the signal specifically localized in some germ cell cytoplasm, principally in SPG (arrow; Figure 3D) and SPC (arrowhead; Figure 3D and inset), indicating a basal rate of autophagy.

In CS, an evident increase of LC3B signal in the cytoplasm of seminoma cells was evidenced (asterisk; Figure 3D and inset) as compared to NP (*p* < 0.001; Figure 3E).

Finally, in LCT, besides the presence of the signal in SPG (arrow; Figure 3D) and SPC (arrowhead; Figure 3D and inset), as found in NP, it also appeared in SPT (dotted arrow; Figure 3D); LC3B fluorescence signal was increased (*p* < 0.01; Figure 3E) as compared to NP.

## 4. Discussion

In this paper, with the aim to expand the knowledge on the biology of CS and LCT, we analyzed the expression and localization of PTMA, not only for the first time in human testis, but also hypothesizing its putative involvement in TCs.

Indeed, if on one hand, the role of PTMA, as a consequence of its proliferative properties, in the insurgence/progression of several cancers has been extensively demonstrated [9,10,11,12,13,14], on the other, just a few papers described its function in the differentiative processes leading to spermatozoa production [25]. In our previous works, we proposed a role for PTMA in the meiotic and post-meiotic phases of spermatogenesis, due to its involvement in the acrosome biogenesis and acrosome-nuclear docking, as a result of its interaction with perinuclear theca proteins [28]. Moreover, we suggested a fascinating function for PTMA, not yet confirmed, in the fertilization events, because of its localization in the inner acrosome membrane in both rat and human sperm [29]. Indeed, PTMA shows high efficacy in decondensing human sperm chromatin; thus, following its entry into ooplasm, it may participate in pronuclear chromatin decondensation [16].

This paper provided two novelties concerning PTMA: (1) the first evidence of its expression and localization in human testis and (2) its increased protein levels in two of the most common TC: CS and LCT.

The specific localization of PTMA in SPC and SPT also in human testis not only supports the hypothesis of its involvement in spermatogenesis, but also the phylogenic importance of this polypeptide, whose role has been established ranging from non-mammalian vertebrate to humans.

Regarding PTMA and PCNA presence in the cytoplasm and the nucleus of cells in TC samples, we confirm other studies, reporting the increased PTMA expression, together with *c-myc*, another cell proliferation marker, in several human cancers and cell lines [19]. Thus, the proliferative and antiapoptotic properties of PTMA may contribute to the cell cycle progression also in TC. PTMA, because of its intrinsically disordered structure, can interact with many partners, both at the nuclear (as histone H1) [15,16,17,18] and cytoplasmic level. In this case, it has been reported that PTMA interacts also with apoptotic factors, as ANP32A, inhibiting the apoptosome formation [50,51]. However, we should also consider that, when apoptosis occurs, caspase-3 cleaves PTMA, generating a truncated, inactive form (aa 1–100) and the C-terminal fragment. Thus, we can hypothesize that, as in CS and LCT, apoptosis is downregulated, and there is no, or little, PTMA cleavage by caspase-3.

It is worth remembering that several pieces of experimental data proved that PTMA expression was enhanced following estrogen stimulation [52,53,54]. Moreover, in both CS and LCT, estrogen signaling is enhanced, for the overexpression of GPR30 [55,56,57,58,59], as well as for the increased expression of aromatase, the enzyme converting testosterone into estradiol [60,61,62,63], resulting in increased estradiol concentration [63,64] and, consequently, disturbing the hormonal milieu. Thus, the enhanced estrogen signaling, eliciting PTMA expression, may lead to stimulate cell proliferation.

In addition, the altered hormonal environment may have another consequence on PTMA: indeed, in LCT samples, we also detected a specific localization signal in SPC nucleus that, on the contrary, was not visible in NP. In our previous paper, we found a comparable situation concerning the formin DAAM1 [65], a protein that was believed to be exclusively cytoplasmic [66,67], but that can be shuttled in the nucleus on the condition of stimulated germ cells proliferation [40,65]. Thus, we can speculate that, also in this case, the enhanced testosterone production and secretion may promote PTMA shuttling to the SPC nucleus to favor their proliferation. Further studies are needed to verify this point.

It is worth noting the crosstalk between apoptosis and autophagy during cancer progression. Noticeably, apoptosis downregulation is one of the most typical causes/manifestations of cancer; on the contrary, autophagy plays a double-edged sword in cancer biology: in the initial stages of cancer progression, it removes damaged cytosolic organelles and contents (as mutated DNA), acting as tumor suppressor [68]; conversely, autophagy can promote cancer cells growth and progression, functioning as an adaptive metabolic response, through recycling macromolecules, in hostile conditions, as poor vascularization, nutrient deprivation, and hypoxia in solid tumors [69].

In this context, considering that PTMA inhibits caspase activation and, consequently, may promote the switch from apoptosis to autophagy, the increased autophagic rate in CS and LCT samples, as showed in this paper, led us to hypothesize a putative role for PTMA as an anti-apoptotic and pro-autophagic factor.

## 5. Conclusions

In conclusion, the first evidence of PTMA expression and localization also in the human testis is reported here, and its increased protein level in two TC, CS and LCT. In addition, our results corroborate previous reports demonstrating the anti-apoptotic action of PTMA and, also, suggest it may act as a switcher from apoptosis to autophagy, to preserve and maintain the cell survival in the adverse environment in which cancers develop.

Although this study is preliminary, mainly due to the reduced number of the used samples, it highlighted interesting new insights into the CS and LCT biology, also supporting the significant role of PTMA in regulating cell cycle progression occurring before, during, and after normal and/or pathological cell differentiation. However, more studies are needed to validate all the results above.

## Data Availability

The authors confirm that the data supporting the findings of this study are available within the article.

## Supplementary Materials

The following supporting information can be downloaded at: https://www.mdpi.com/article/10.3390/biom12091210/s1, Figure S1: Hematoxylin-eosin staining of normal and pathological testicular tissues.
