# Supplementary material for: First Evidence of the Expression and Localization of Prothymosin α in Human Testis and Its Involvement in Testicular Cancers
Source: Biomolecules. 2022 Aug 31;12(9):1210. doi: 10.3390/biom12091210 (PMC9496091; doi:10.3390/biom12091210)
Supplement: Supplementary file 1 [file biomolecules-12-01210-s001.zip › biomolecules-1858024-supplementary.pdf]

Histology of non-pathological (NP), classic seminoma (CS) and Leydig cell tumor (LCT) testicular tissue.
In Figure S1 are shown representative images of histological features of non-pathological (NP) testicular tissues and of two TCs, CS and LCT.

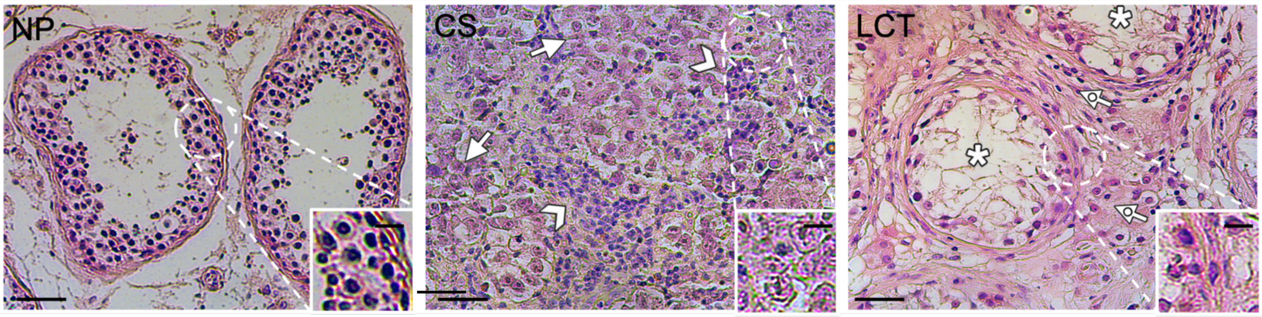

Figure S1. Hematoxylin-eosin staining of normal and pathological testicular tissues. Evaluation of testicular histology of non-pathological (NP), classic seminoma (CS), and Leydig cell tumor (LCT) samples. Arrow: seminoma cells; arrowhead: lymphocytic infiltration; dotted arrow: interstitial cells with abundant, eosinophil cytoplasm; *: testicular parenchyma; Scale bars represent 20 μm and 10 μm in the insets.

In NP it is possible to see that both germ and somatic cells present the normal morphological characteristics, contributing to the normal spermatogenesis.
CS is the most common among germ cell tumor (GCT; about 90% of cases) and it is characterized by unvaried distribution of similar, rounded cells with large, centralized nuclei and nucleoli (arrows; Fig. S1 and inset, CS) and by abundant lymphocytic infiltration in the stoma (arrowhead; Fig. S1, CS).
LCT represents 1% to 3% of sex cord–gonadal stromal tumors (SCGST) and, in these samples, the testicular parenchyma is limited at the periphery of the tissue (asterisk; Fig. S1, LCT), and it was surrounded by an evident monomorphic proliferation of tumoral Leydig cells (LC; dotted arrow; Fig. S1 and inset, LCT).
